# Measuring urofecal glucocorticoid metabolites in broiler chicken: a noninvasive tool for assessing stress as a marker of welfare

**DOI:** 10.1016/j.psj.2024.104162

**Published:** 2024-08-03

**Authors:** Tanja E. Wolf, Kathrin Toppel, Lea Jacobsen, Robby Andersson, Chadi Touma

**Affiliations:** ⁎ Department of Behavioural Biology, Osnabrück University, 49076 Osnabrück, Germany; † Mammal Research Institute, University of Pretoria, 0002 Pretoria, South Africa; ‡ Department of Applied Poultry Sciences, University of Applied Sciences Osnabrück, 49090 Osnabrück, Germany

**Keywords:** urofecal glucocorticoid metabolite, corticosterone, production animal, broiler, stress physiology

## Abstract

The poultry industry is an important and still growing sector in many parts of the world. For ethical reasons and due to increased consumer awareness for animal welfare in production animals, it is of importance to establish a reliable and objective test system for monitoring and improving health and welfare. During the rearing process, broiler chickens are exposed to numerous potential stressors and management interventions (e.g. weighing of individual animals, preslaughter fasting and capture processes), but assessing the level of stress perceived by the animals entirely through behavioral observations can be challenging. Monitoring stress-related physiological markers, such as glucocorticoids, can be an accurate and presumably more objective addition. To avoid additional stressors induced by blood collection, a noninvasive approach using urofecal samples is advisable. However, a thorough validation is needed to establish a suitable test system for measuring stress hormone levels, including potential effects of the time of day of collection or the time that has elapsed since defecation.

Therefore, the aim of this study was to test the stability of urofecal glucocorticoid metabolites (**ufGCM**) postdefecation, to determine time of day effects on ufGCM levels, and to investigate the effect of standard management procedures on ufGCM concentrations in broiler chickens.

Our results revealed a time window of 4 h in which fecal samples from broilers can be collected without major alterations to the ufGCM concentrations. In this regard, a “fecal box” proved useful for collecting uncontaminated fresh samples. The time of day of sample collection did not influence ufGCM concentrations significantly. Moreover, the used assay proved to be sensitive enough to detect even small and short-lasting activations of the HPA axis induced by handling, confinement, and fasting. Thus, the system used can be a powerful and easy to apply tool in a chicken production setup for assessing stress as a marker of welfare in commercially housed broiler chickens, which in the long-term can also improve production, particularly with regard to process quality.

## INTRODUCTION

The poultry industry is an important and still growing sector in many parts of the world (FAO, 2023). For ethical reasons and due to increased consumer awareness for animal welfare in production animals (Sans et al., 2014), it is in the interest of consumers as well as farmers to improve the housing conditions of production animals and to establish a reliable and objective test system for assessing and monitoring health and welfare of broiler chickens. The German Animal Welfare Act obliges livestock farmers to collect and evaluate animal welfare indicators. The aim is to check whether the animals are fed, cared for and housed in a way that is appropriate to their needs and whether they have opportunities for species-appropriate activity. The welfare quality protocol (**WQP**) for poultry provides one of several systems available to assess broiler welfare (WQ, 2009) during the rearing process. Therefore, on-farm, it is common to evaluate the welfare of broiler chickens using indicators like mortality and foot pad condition (Hunter et al. 2017). However, several measurements taken with the WQP have been found to be rather time consuming as well as subjective to the observer (Blatchford 2017; Buijs et al, 2017). In recent years however several attempts to improve the protocol have been made (deJong et al. 2016). A complementary approach would be the monitoring of stress-related physiological markers, such as glucocorticoids (**GC**) as a presumably more objective addition to other markers of welfare. Housing or management factors associated with stress responses could also be identified and minimized or adjusted in order to improve animal welfare. During the rearing process, commercial broiler chickens are exposed to numerous potential stressors and management interventions like weighing of individuals, feather scoring, or preslaughter fasting and capture processes (Kannan and Mench, 1996). Especially the preslaughter period can be perceived as stressful due to the changes in the environment and climatic conditions, the food deprivation, and sometimes pain or anxiety inflicted by the handling (Terlouw et al, 2008)

When confronted with a stressor the hypothalamus releases corticotropin-releasing hormone (**CRH**), which stimulates the pituitary to release adrenocorticotropic hormone (**ACTH**) that in turn stimulates the adrenal cortex to release GC (Sapolsky et al., 2000; Palme, 2019). Glucocorticoids are a key element in the neuroendocrine hypothalamic-pituitary-adrenal (**HPA**) stress axis, and their measurement can give insight into an animal's welfare-status (Broom and Johnson, 1993; Palme, 2019). Acute elevations in GC levels can be adaptive, but prolonged elevations can have negative physiological consequences, for example suppression of immune and reproductive functions, as well as growth (Broom and Johnson, 1993; Sapolsky et al., 2000; Hardy et al., 2005). In broilers, it has been shown that prolonged heat stress, and the associated elevated glucocorticoid levels, have negative effects on fat metabolism and muscle growth, and therefore are ultimately negatively affecting the meat quality (Quinteiro-Filho et al, 2012; Nawab et al, 2018). In current poultry rearing systems heat stress is a common problem during the summer months (Del Barrio et al, 2020) and is likely to increase in the light of climate change and global warming. Glucocorticoid concentrations are therefore frequently used as proxies to assess the impact of physiological and environmental stressors.

Blood samples are often used as a matrix for determining GC concentrations in a wide range of species, including chickens. However, this comes with several limitations, such as diurnal variation patterns, but most importantly, it is associated with capture and restraint of the animals for sampling, which is itself perceived as a stressor (Broom and Johnson, 1993; Touma and Palme, 2005). Therefore, a noninvasive method to repeatedly and longitudinally monitor excreted stress hormone metabolites, using urine or feces has become the preferred option (Touma and Palme, 2005; Hodges et al., 2010; Palme, 2012; Palme 2019). Circulating GCs are metabolized by the liver and excreted via the bile into the gut or via the kidneys into the urine (Touma and Palme, 2005). In birds, the urine is excreted together with the feces and often it is difficult or impossible to separate both. However, it is possible to measure steroid hormone metabolites in these so-called urofecal droppings (Bishop and Hall, 1991; Cockrem and Rounce, 1994; Dehnhardt et al., 2003; Rettenbacher et al., 2004). Using droppings as sample material for determining glucocorticoid output in broiler chickens would consequently be an ideal approach, as it does not involve handling and therefore can avoid interference with the results due to handling stress. Additionally, fecal samples are often less affected by diurnal variations in hormone secretion and can be collected comparatively easy (Touma and Palme, 2005).

In unpreserved fecal material the quantity, as well as the composition of fecal glucocorticoid metabolites (**fGCM**) can vary over time, this further degradation is mainly caused by bacterial enzymes in the feces continuing to metabolize GCs, and the resulting metabolites can bind more or less to the antibody of the used enzyme immunoassay (Washburn and Millspaugh, 2002; Touma and Palme, 2005; Heistermann, 2010). Studies in bovines and Japanese quails indicated alterations after a relatively short time frame of 1 to 4 h after defecation (Möstl et al., 1999; Pellegrini et al., 2015), while in domestic pigs fGCM concentrations remained relatively stable over a period of 48 h (Wolf et al., 2020). Similarly, sex steroid metabolite concentrations in domestic fowls remained stable for up to 48 h (Cockrem and Rounce, 1994), while in geese elevated fecal testosterone levels were found after only 3 h post defecation (Hirschenhauser et al., 2005).

Most hormones, including GC, show a diurnal pattern with a peak secretion towards the beginning of the light period in diurnal animals (Möstl and Palme, 2002; Touma and Palme, 2005). This diurnal variation can also be detected in fecal samples of some mammal and bird species, such as mice and geese, but for example in several carnivore species, no such rhythm could be detected (for reviews see Touma and Palme, 2005 and Palme 2019).

Due to the reasons outlined above, a thorough validation is needed in order to establish a suitable test system for the measurement of stress hormone metabolites using urofecal samples of broiler chickens. An enzyme-immunoassay for measuring urofecal glucocorticoid metabolites (ufGCM) has already been established for droppings of laying hens (Rettenbacher et al., 2004; Rettenbacher and Palme, 2009). However, for the use in broiler chickens an extensive validation is still needed, especially when used under commercial farming conditions. The aim of this study was therefore to i) test the stability of urofecal glucocorticoid metabolites post defecation, ii) determine time of day effects on ufGCM levels, and iii) investigate the effect of standard management procedures on ufGCM concentrations in broiler chickens.

## MATERIALS AND METHODS

### Animals and Housing Conditions

Day-old broiler chickens (Ross 308) hatched from the same parent stock were housed in an empty, clean and disinfected barn in 2 equally sized groups (2 pens of 12×1 m) over a rearing period of 41 d. Two independent batches (n = 246 and 240, respectively) were used. The air temperature was kept at 33°C at the chicks' placement and was decreased by 0.5 degrees daily in the first wk, then reduced by 1 degree daily until reaching ∼ 20°C. The broiler chickens were fed a commercially standard diet (3 phase feeding, commercially available starter, grower, and finisher diets) during the rearing period. Feed and water were available *ad libitum*. Chicks were placed on new bedding, 1 kg/m² straw pellet. Broiler chickens were vaccinated at d 0 (IB; hatchery), 13 (ND) and 20 (Gumboro), weighted weekly and foot pads were scored in accordance to Welfare quality (5-point scale; Score 0 – no lesions, Score 4- severe lesions). After 24 h of light, from d 2 the lighting program was 8 h dark (10 pm to 6 am) and 16 h light (6 am to 10 pm). All management aspects were in accordance with the national requirements for broiler husbandry (TierSchNutztV, 2021).

### Sample Collection

**Figure 1:**
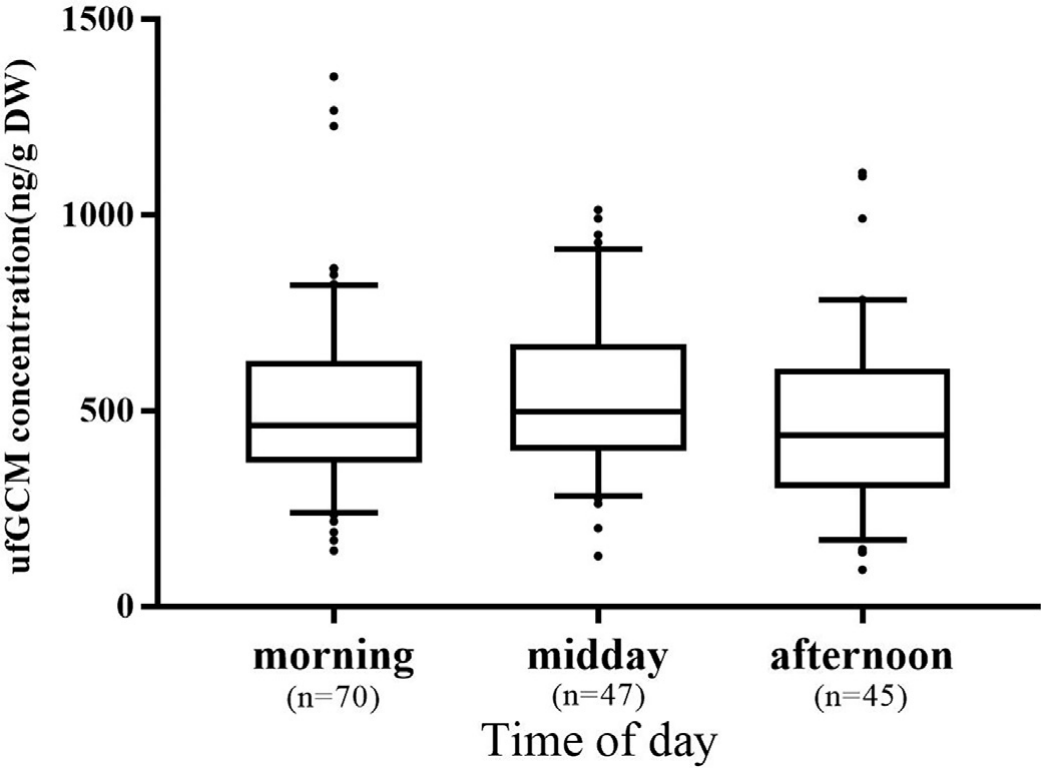
Concentration of urofecal glucocorticoid metabolites (ufGCM; ng/g dry weight [DW]) at 3 different sampling times (morning: 2–4 h after the start of the daily light regime, midday: 6–8 h after the start of the daily light regime, afternoon: 10–12 h after the start of the daily light regime).

Urofecal samples were collected using a so-called ‘fecal boxes’ consisting of a 50 × 50 × 10 cm plastic container with a metal mesh (20 × 20 mm spaces) on top. The broiler chickens voluntarily sit and also partly sleep on the boxes and the feces fall through the mesh into the container, where they can be collected without contamination from surrounding litter. In order to assist the chicks to access the box metal ramps were added in the first 3 wk of the rearing period (see Figure 1 Supplementary Material). The floor of the ‘fecal box’ was covered with conventional parchment paper to avoid sticking. For the main study, pooled samples (1–4 individual samples), from the boxes were collected at preset times in plastic bags and stored at -20°C until analysis. The samples were collected within 4 h of defecation, as evaluated by the results of the degradation study detailed below.

Additionally, litter samples were collected from the floor of the housing pens at different time points during the rearing period (d 0, 2, 9, and 40) and were tested in the enzyme-immunoassay to evaluate the potential effect of litter attached to droppings to simulate urofecal sample collection without a “fecal box” (the results of this experiment are presented in the supplementary material).

### Effect of Collection Time and Stability Test

To investigate a possible influence of the time of collection on the ufGCM concentrations over the course of the rearing period, urofecal samples were collected at 3 different diurnal time windows (morning: 2 to 4 h after the start of the daily light regime, midday: 6 to 8 h after the start of the daily light regime, afternoon: 10 to 12 h after the start of the daily light regime).

In order to determine possible effects of the time since defecation on the measured ufGCM concentrations, in total 64 freshly voided samples were collected between d 2 and 40 of the rearing period. In total 8 independent test lines were created, 1 test line consisted of 8 of the 64 samples that were then thoroughly homogenized and divided again into 8 equally sized subsamples, forming 1 test line. This procedure was done 8 times, resulting in 8 independent test lines. This pooling of samples ensures that all subsamples in each of the 8 test lines have an equal distribution of ufGCM and a comparable size. All subsamples then were stored under standard conditions (22°C, 60% humidity). At different time points (0, 1, 2, 4, 8, 12, 24, and 48 h) 1 subsample from each of the 8 test lines was frozen at -20°C until analysis.

### Impact of Management Procedures

During the normal rearing period, broilers were weekly subjected to routine checks, which included body weight measurements and scoring of the foot pad conditions. To determine the effect of these routine procedures on ufGCM concentrations as a biological validation, samples from animals directly involved (handled individuals) and not involved (nonhandled individuals) in these procedures were collected prior, during and post procedure (every 2 h for a period of 8 h), and were compared to baseline ufGCM levels. Baseline levels were calculated from samples collected over a period of 5 to 10 d prior to the interventions in order to establish a reliable baseline.

Additionally, the effect of the 12 h feed fasting period before slaughter (earliest possible date regarding German Legislation) was investigated and compared with a capture and transport procedure. The capture procedure took place in the morning of the intervention day and lasted for approximately 30 minutes. The animals were then put into standard transport crates, where they remained for 2 h. A transport was simulated by carrying the crates around. Afterwards the animals were released back into the housing pens and sample collection took place every 2 h for a period of 8 h.

All sampling and handling procedures took place during normal rearing operations and corresponded to the requirements, §19 (1), of the TierSchNutztV (2021).

### Sample Preparation and Analysis

In the lab, the samples were freeze-dried for approximately 50 h (Alpha LSCbasic, Christ, Germany), pulverized, and sieved through a thin strainer (2 mm) for removal of fibrous material. For steroid extraction, 0.05 g of fecal powder was vortexed (Multitube vortexer, Thermo Fisher Scientific, Germany) for 30 minutes with 3 ml of 60% methanol. After centrifugation for 15 minutes at 2500 g, the supernatants were aliquoted and stored at -20°C until analysis.

Urofecal glucocorticoid metabolite concentrations were determined using a group-specific cortisone enzyme-immunoassay (antibodies raised in rabbits against 4-pregnene-17α,21-diol-3,11,20-trione-21-HS), which has been established and validated for measuring ufGCM in chickens (Rettenbacher et al., 2004; Rettenbacher et al., 2009).

Inter-assay coefficients of variation, determined by repeated measurement of high- and low-value quality controls, were 12.4 and 15.8 %, respectively. Intra-assay coefficients of variation were 13.1 % for low and 9.1 % for high quality controls. All samples were analyzed in duplicates as previously described (Ganswindt et al., 2002).

### Statistical Analysis

To evaluate the effect of the time of day of sampling on ufGCM concentrations, results from the 3 sampling time points were compared using the Kruskal-Wallis H-test. The relative change of ufGCM concentrations at different time points post defecation (n = 8 samples at each time point) was calculated using the mean value determined at t = 0 as 100%.

The increase of ufGCM concentrations in animals subjected to the routine management procedures were evaluated using the Wilcoxon rank sum test. The effect of the fasting period and stay in the transport boxes were described as percent changes from baseline levels.

All statistical analyses were performed using R, version 4.0 (R Core Team, 2013). The figures were created with the software GraphPad Prism (version 7.05.237, La Jolla, CA).

## RESULTS

### Animal-Related Parameters

The cumulative mortality rates for the 2 batches were 3.7 % (n = 9) and 2.9 % (n = 7), respectively, and feed conversion ratio was 1:1.54 and 1:1.60, respectively. Live weight was in accordance to the Ross performance guide (Aviagen, 2022) and the average weight of all birds in the flock at d 41 was 3,011g (±324g) and 2,989g (±301g), respectively. Foot pad condition was inconspicuous, with 95% of all feet scored without any lesion (score 0) and 5 % with slight lesions (score 1) a day before slaughtering.

### Effect of Collection Time and Stability Test

In order to investigate the influence of the collection time on ufGCM concentrations in the feces of broilers, samples were collected at different times of day and compared with each other. Samples were collected at 3 time windows (morning, midday, afternoon) throughout the rearing process. No significant influence of the time of collection on ufGCM concentrations was found (Kruskal–Wallis H-test, chi-squared = 2.047, df = 2, *P* = 0.359; Figure 1).

**Figure 2:**
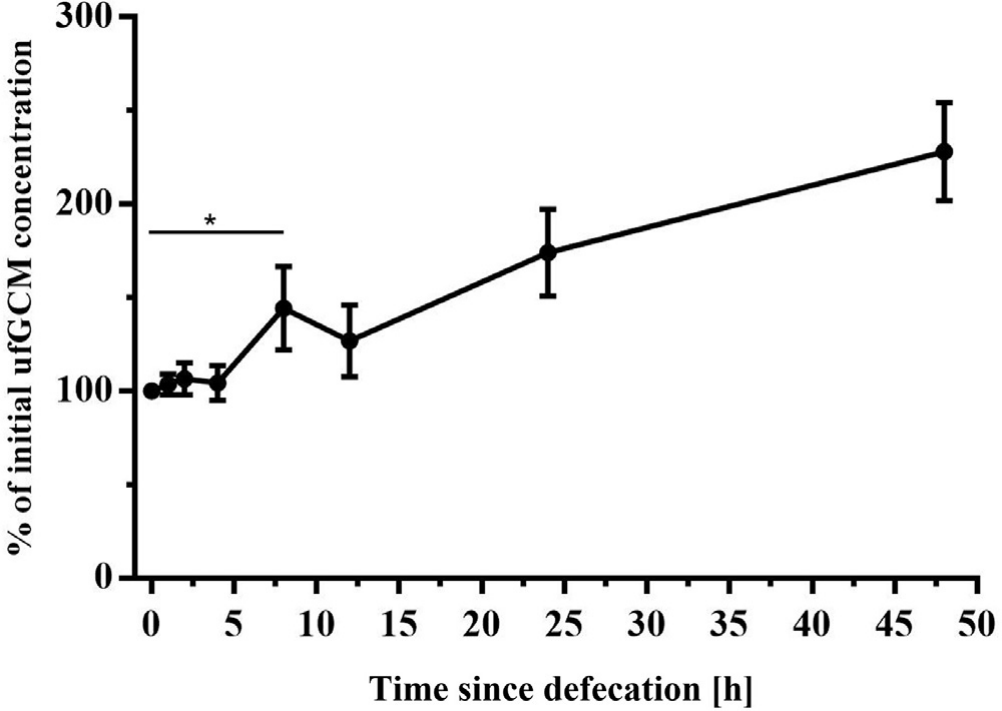
Relative change (percent (mean ± SE)) of immunoreactive urofecal glucocorticoid metabolite (ufGCM) concentrations at different times after defecation and incubation of the fecal samples under normal housing climate conditions (22°C, 60% humidity, time points: t = 0, 1, 2, 4, 8, 12, 24, and 48 h)

The experiment investigating the stability and degradation of stress hormone metabolites revealed statistically significant differences in ufGCM concentrations over time, when incubated samples were compared to fresh samples (Kruskal–Wallis H-test F = 7.103, df = 7, *P* < 0.001, Figure 2). Within the first 4 h, the concentration remained relatively stable with a variation of only ± 5%. After 8 h, however, there was a significant increase in the measured ufGCM concentration by an average of 44% (Figure 2).

### Impact of Management Procedures

**Figure 3:**
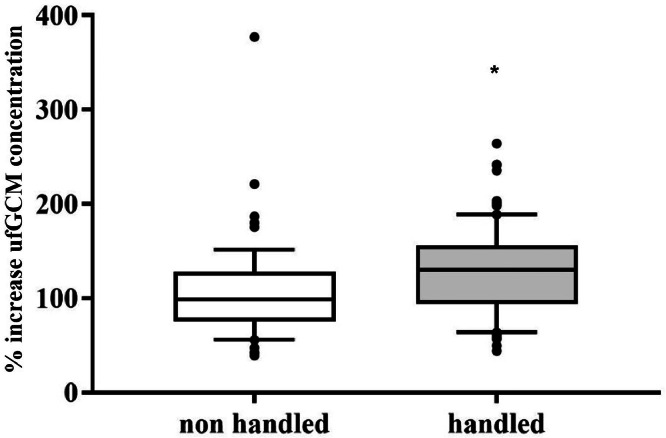
Relative change of urofecal glucocorticoid metabolite (ufGCM) concentrations after handling for routine health checks (weighing, plumage inspection and foot pat scoring) of broiler chicken.

During routine health checks, such as weighing and scoring of foot pad conditions, for bystander animals that were not caught or handled, no significant increase of ufGCM concentration was detected (Wilcoxon rank sum test, Z = 926, *P* = 0.630). In contrast, in the treated animals, a statistically significant effect of the routine control checks on ufGCM concentrations was observed, although the increase was relatively small (about 50% above baseline, Wilcoxon rank sum test, Z = 1,164, *P* = 0.001; Figure 3) and the ufGCM concentration returned to baseline levels within 12 h after the procedure.

**Figure 4:**
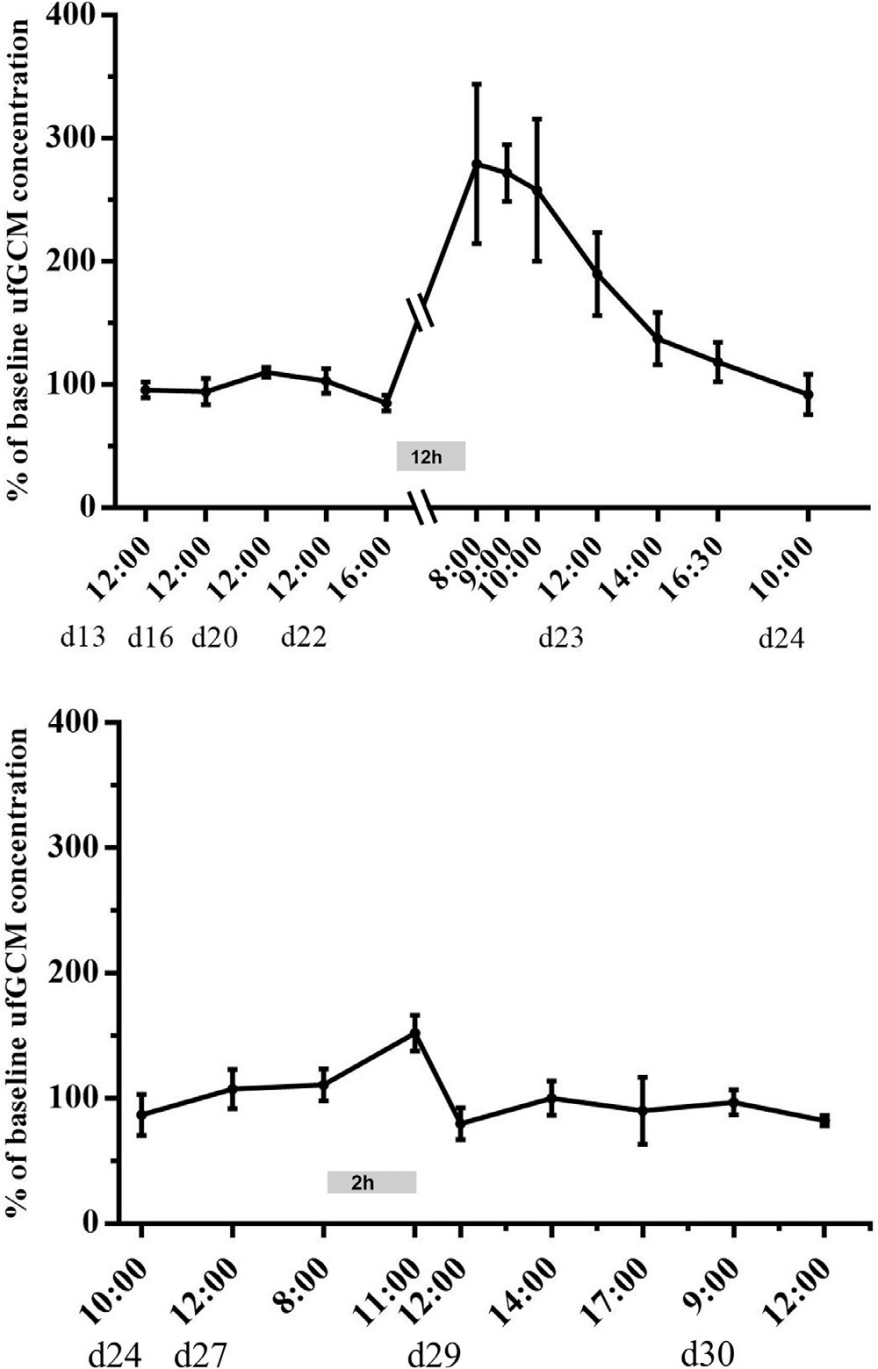
Time course of relative change (percent (mean ± SE)) of urofecal glucocorticoid metabolite (ufGCM) concentrations of broiler chickens (age: 13–30 d) subjected to a 12 h preslaughter fasting period (A) or capture and 2 h confinement in transport boxes (B). Grey boxes in the panels indicate the timing of the respective procedures.

Moreover, the catching procedure and the preslaughter feed fasting at the end of the rearing period had a significant impact (Figure 4). A clear increase of the ufGCM concentration of approx. 200% was observed after the 12 h fasting period (Figure 4A), while the capture and the 2 h confinement in the transport boxes caused an increase of ufGCM concentrations of approximately 50% (Figure 4B), which was comparable to the routine checks during rearing (Figure 3). Here too, ufGCM values returned relatively quickly, reaching baseline levels at the end of the intervention day.

## DISCUSSION

Reliable monitoring of adrenocortical activity as a physiological marker to evaluate animal welfare requires a solid validation of the test system, especially when using fecal samples as a matrix. The gastrointestinal transit time (**GIT**) and the metabolism of steroids can differ from species to species and can therefore lead to the excretion of different glucocorticoid metabolites (Palme et al., 1996; Touma and Palme 2005; Palme, 2019). Previous studies used physiological (ACTH challenge) as well as a biological validation experiments (transport and restraint) to establish a suitable enzyme-immunoassay measuring ufGCM in laying hens (Rettenbacher et al., 2004; Rettenbacher and Palme, 2009). In the current study, we tested the suitability of this assay for broiler chickens using different routine management procedures as biological validation interventions. Thus, we aimed to establish a suitable and practical test system enabling sample collection and valid ufGCM evaluation under commercial housing conditions.

### Effect of Collection Time and Stability Test

In most vertebrate species plasma glucocorticoids have a distinct diurnal pattern (Sapolsky et al., 2000; Touma and Palme, 2005; Palme, 2012)*.* In laying hens, a diurnal rhythm could be found with highest levels at the end of the dark phase (Beuving and Vonder, 1977), whereas in broiler chicken an increase in plasma corticosterone concentrations could be shown in the beginning of the light phase (de Jong et al., 2001). However, in our study no significant influence of the time of collection on the ufGCM concentration in the broiler chicken was found (see Figure 1). This stands in line with findings in laying hens, where also no diurnal pattern was seen in feces (Rettenbacher et al., 2004). Using fecal samples as a matrix therefore seems to be of advantage, especially when a frequent sampling at a specific time of day cannot be guaranteed.

In order to evaluate possible changes in hormone metabolites detected in unpreserved samples, the stability of ufGCM postdefecation was determined. Concentrations remained relatively stable with a variation of only 5% within the first 4 h, followed by a clear increase of 44% (see Figure 2). A similar pattern of increasing urofecal glucocorticoid metabolite levels after more than 4 h have been reported in Japanese quails (Pellegrini et al. 2015). In geese, elevated fecal testosterone levels were found as soon as 3 h after defecation and storage at room temperature (Hirschenhauser et al., 2005). These changes in steroid metabolite concentrations are driven by the continued microbial metabolism of steroids in the feces, which could lead to either increased or decreased immuno-reactivity in the samples, i.e. detection of more or less steroid metabolites by the antibodies used in the assays.

Although it is recommended that fecal samples are collected and frozen as soon as possible after defecation, our results indicate that a 4 h time window can be regarded safe for collection without negative effects on immunoreactive ufGCM concentrations. This is especially important as a regular and immediate sampling is difficult in spacious stables under commercial housing conditions.

In this regard, the "fecal box" has been proven useful for collecting chicken droppings. Any litter residues (fresh material) sticking to the feces had no effect on the measured ufGCM concentrations measured with the enzyme immunoassay. However, litter contaminated with feces (old material) can significantly influence the measurement result as old litter is contaminated with fecal materials and can therefore falsely increase ufGCM concentrations (see Figure 1 in the Supplementary Material). A fecal box, as used in our study, can help to prevent such contamination of fresh samples with old fecal material and thus makes the results much more reliable.

### Impact of Management Procedures

When not directly involved in management procedures, such as weighing and foot pad scoring, individuals showed no significant elevation of ufGCM concentrations. In contrast, animals that have been handled and scored showed a small, but significant increase in ufGCM concentrations (see Figure 3). Similar effects were found in restraint cockerels and broiler chickens subjected to different handling methods (Kannan and Mench, 1996; Heiblum et al., 2000), although Rettenbacher and Palme (2009) did not find an increase in ufGCM concentrations after a 10-minute restraint of laying hens. Interestingly, the method of handling can cause significant differences, with animals being held in an inverted position showing almost doubled corticosterone concentrations compared to animals being held upright (Kannan and Mench, 1996). In our study, the animals were all handled in an upright position, i.e. a smaller and shorter increase in plasma corticosterone was expected (Kannan and Mench, 1996; Rettenbacher and Palme, 2009). In a commercial setting, it can be expected that the handling procedures would be less gentle, depending on the type of handling used by the farm personnel, thus most likely resulting in a higher physiological stress response. Nevertheless, even the small increase induced by the brief health check manipulation in our study could be monitored in the ufGCM concentrations, showing the high sensitivity of the test system.

A larger impact on ufGCM concentrations was found during longer confinement (2 h in transport boxes) and food deprivation (12 h preslaughter fasting) (see Figure 4)*.* In laying hens, higher plasma corticosterone levels were found in animals that were crated and transported for 2 h compared to animals only being handled and removed from their home cages (Broom et al., 1986). In broilers, also the crating process seemed to have a higher impact than the handling per se (Kannan and Mench, 1996). However, the most profound impact on ufGCM concentrations in our study was found after the 12 h preslaugther fasting process (see Figure 4A). Elevated plasma corticosterone levels have been reported in chickens that had no access to food for up to 24 h, as well as in chickens under longer food restrictions (Scanes et al., 1980; Kannan and Mench, 1996; Yan et al., 2021). This strong physiological stress response can be explained by the fact, that over the last century, broiler chickens have been bred specifically to reach a higher weight in a shorter timeframe, leading to an increased appetite and a subsequent increased voluntary feed intake (for a detailed review see Tallentire et al., 2016). The fasting procedure therefore likely puts a higher pressure on these high metabolizing animals. An additional psychological factor has been proposed by Kannan and Mench (1996), as in most commercial settings, the feeders are only pulled out of reach of the animals and not taken out of the pens, i.e. are remaining still in sight. This potential additional stress effect of food visibility on the chickens should be investigated in future studies.

However, after all procedures assessed in our study, ufGCM concentrations returned to baseline within 12 h. This is consistent with a study on laying hens that were subjected to transport, as well as to short term restraint (Rettenbacher and Palme, 2009).

In conclusion, our study evaluated a time window of 4 h in which fecal samples from broilers can be collected without relevant alterations to the ufGCM concentration. In this regard, it was shown that the ‘fecal box’ is as a useful method to collect uncontaminated fresh fecal samples. Moreover, the used cortisone assay system proved to be sensitive enough to detect substantial, but also small and short-lasting activations of the HPA axis in broiler chickens. Thus, in addition to other welfare indicators, such as behavioral observations or physical condition, the system implemented here can be a powerful and easy to apply tool in a commercial setup for assessing stress as a marker of welfare in commercially housed broiler chickens, which in the long term can also improve production, particularly with regard to process quality.

## DISCLOSURES

The authors declare the following financial interests/personal relationships which may be considered as potential competing interests: Tanja E. Wolf, Kathrin Toppel, Lea Jacobsen, Robby Andersson, Chadi Touma reports financial support was provided by Lower Saxony State Ministry of Science and Culture. Chadi Touma reports financial support was provided by Evonik Operations GmbH. If there are other authors, they declare that they have no known competing financial interests or personal relationships that could have appeared to influence the work reported in this paper.

## Appendix Supplementary materials

Image, application 1

Image, image 2

Image, image 3

## Footnotes

Supplementary material associated with this article can be found in the online version at doi:10.1016/j.psj.2024.104162.
