# Supplementary material for: Measuring urofecal glucocorticoid metabolites in broiler chicken: a noninvasive tool for assessing stress as a marker of welfare
Source: Poult Sci. 2024 Aug 3;103(11):104162. doi: 10.1016/j.psj.2024.104162 (PMC11381796; doi:10.1016/j.psj.2024.104162)
Supplement: Supplementary file 1 [file mmc1.docx]

**SUPPLEMENTARY MATERIALS**

The so called ‘fecal box’ can help collecting fresh fecal samples without contamination with old fecal material. In order to assist the chicks to access the box, metal ramps were added in the first 3 weeks of the rearing period Suppl. Fig. 1).

Litter samples collected from the floor of the housing pens at different time points during the rearing period were tested in the assay for assessing the effects of a possible cross-contamination of the droppings. Fresh litter samples did not influence the measurements in the enzyme-immunoassay (EIA). However, with feces contaminated litter samples showed elevated concentrations (Suppl. Fig.2).
