# Supplementary figures and images for: Measuring urofecal glucocorticoid metabolites in broiler chicken: a noninvasive tool for assessing stress as a marker of welfare
Source: Poult Sci. 2024 Aug 3;103(11):104162. doi: 10.1016/j.psj.2024.104162 (PMC11381796; doi:10.1016/j.psj.2024.104162)

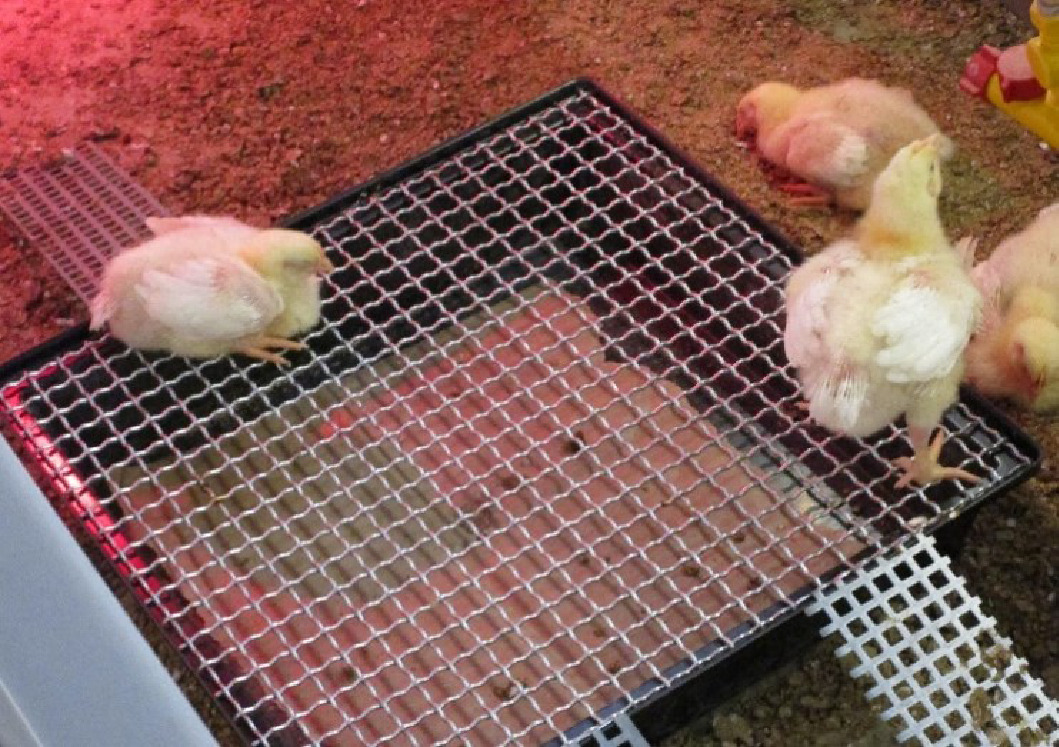

Supplement: Supplementary file 2 [file mmc2.jpg]

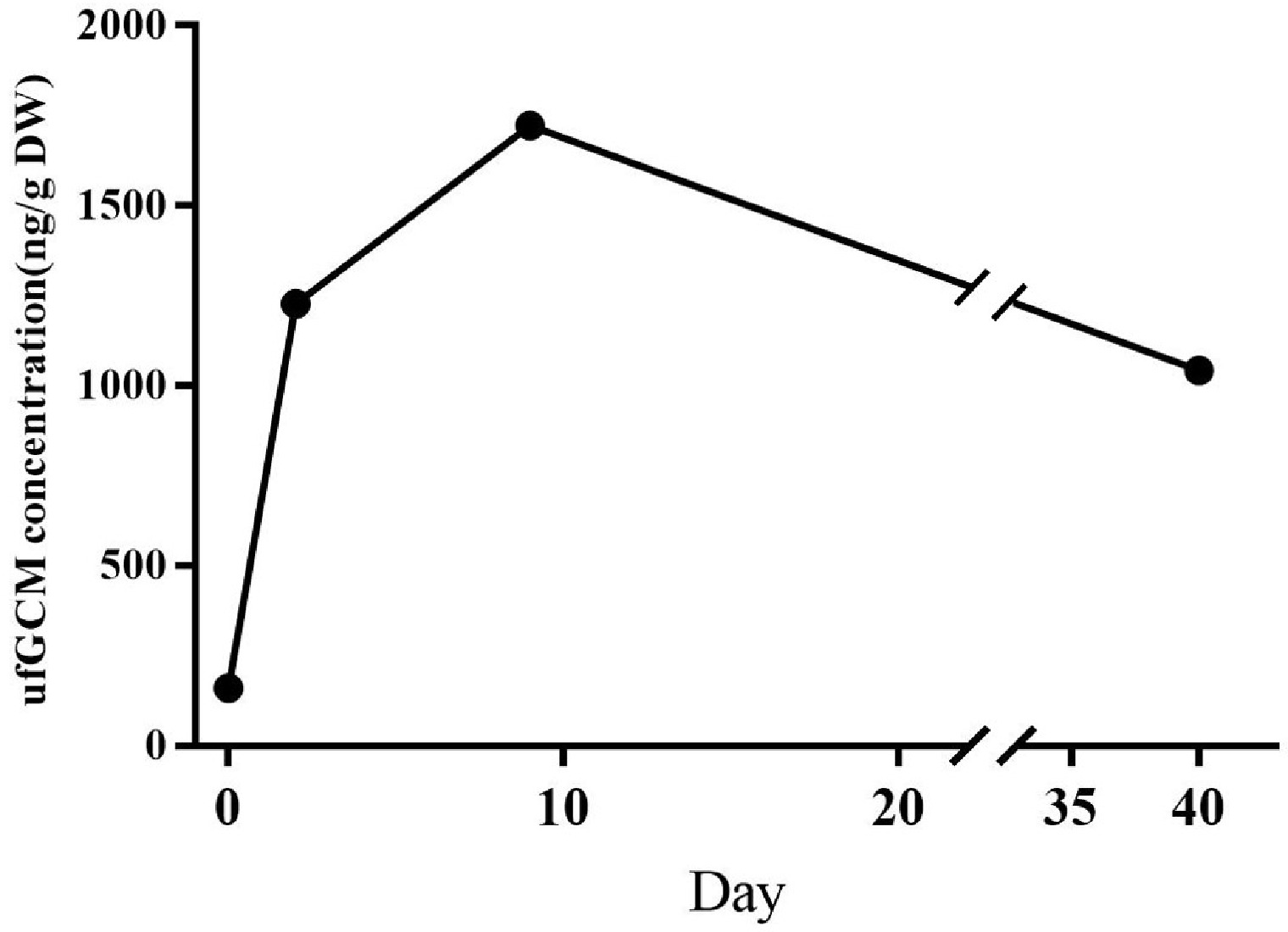

Supplement: Supplementary file 3 [file mmc3.jpg]
